# Magnetic-targeting of polyethylenimine-wrapped iron oxide nanoparticle labeled chondrocytes in a rabbit articular cartilage defect model†

**DOI:** 10.1039/c7ra12039g

**Published:** 2018-02-16

**Authors:** Xiaoyuan Gong, Fengling Wang, Yang Huang, Xiao Lin, Cheng Chen, Fuyou Wang, Liu Yang

**Affiliations:** Center for Joint Surgery, Southwest Hospital, Third Military Medical University (Army Medical University) 30 Gaotanyan Main St. Shapingba Dist. Chongqing 400038 PR China sliegxy@gmail.com wlsd3300@126.com 279077061@qq.com 502048466@qq.com cclljjff@163.com wfy731023@163.com jointsurgery@163.com

## Abstract

Osteoarthritis (OA) is the most prevalent form of joint disease and lacks effective treatment. Cell-based therapy through intra-articular injection holds great potential for effective intervention at its early stage. Despite the promising outcomes, major barriers for successful clinical application such as lack of specific targeting of transplanted cells still remain. Here, novel polyethylenimine-wrapped iron oxide nanoparticles (PEI/IONs) were utilized as a magnetic agent, and the *in vitro* efficiency of PEI/ION labeling, and the influence on the chondrogenic properties of chondrocytes were evaluated; the *in vivo* feasibility of magnetic-targeting intra-articular injection with PEI/ION labeled autologous chondrocytes was investigated using a rabbit articular cartilage defect model. Our data showed that chondrocytes were conveniently labeled with PEI/IONs in a time- and dose-dependent manner, while the viability was unaffected. No significant decrease in collagen type-II synthesis of labeled chondrocytes was observed at low concentration. Macrographic and histology evaluation at 1 week post intra-articular injection revealed efficient cell delivery at chondral defect sites in the magnetic-targeting group. In addition, chondrocytes in the defect area presented a normal morphology, and the origin of cells within was confirmed by immunohistochemistry staining against BrdU and Prussian blue staining. The present study shows proof of concept experiments in magnetic-targeting of PEI/ION labeled chondrocytes for articular cartilage repair, which might provide new insight to improve current cartilage repair strategies.

## Abstract 2

Magnetic-targeting outcome in the knee joint of experimental rabbit model at 1 week post intra-articular injection.

## Graphical abstract

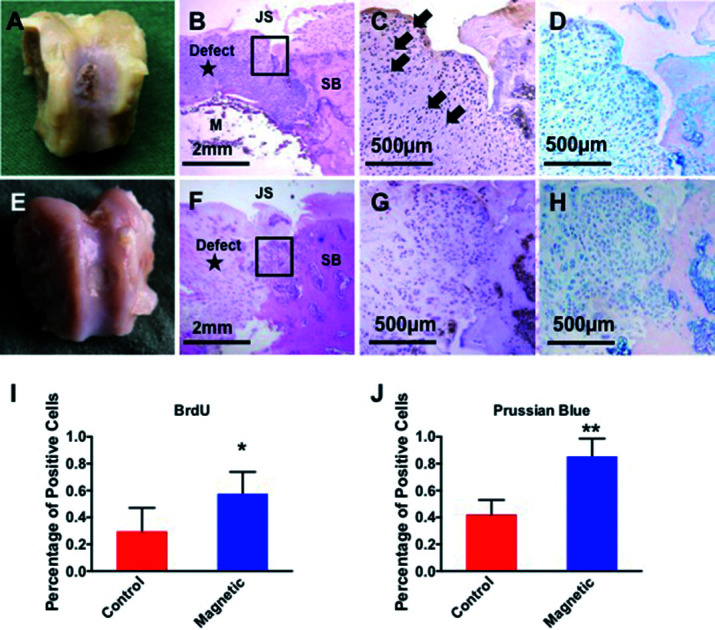

## Introduction

Osteoarthritis (OA) is the most prevalent form of arthritis and a leading cause of disability, and its etiology is still largely unknown. Current treatments for OA are mainly palliative until the joints become totally dysfunctional and prosthetic replacements become necessary. In order to prevent OA progression, effective intervention is needed at its early stage. Articular cartilage is an avascular, aneural, and relatively acellular connective tissue with low metabolic activity and limited self-healing response.^1^ Since articular cartilage does not regenerate when it is degraded, cell-based therapy such as stem cells or autologous chondrocytes implantation holds promise for the repair of chondral defects to achieve the regeneration to hyaline cartilage. Compared with conventional cell delivery methods, intra-articular injection has been suggested as convenient, beneficial, and less invasive by recent clinical studies.^2,3^ Despite these promising outcomes, major barriers for successful clinical application such as lack of specific targeting of transplanted cells due to the complex anatomical structure and physiological environment still remain.

The use of iron oxide nanoparticles (IONs) for *in vivo* cell tracking has been proved promising in recent years.^4,5^ In order to develop a non-invasive technique for *in vivo* detection of transplanted cells, IONs was internalized into cells prior to transplantation *via* endocytosis and served as contrast agent in numerous studies.^4–7^ Intracellular iron oxide reduces local magnetic field homogeneity, allowing the cells to be visualized as signal voids with magnetic resonance imaging (MRI).^4^ The *in vitro* efficacy and safety of using IONs magnetic-labeling has been studied by previous studies.^7,8^ In addition, the feasibility of *in vivo* tracking of IONs-labeled chondrocytes and bone marrow derived stem cells (BMSCs) with MRI has also been demonstrated by our previous studies using large animal models.^4,5^ Moreover, the magnetic properties of IONs allow it to be manipulated mechanically by a magnetic field gradient, which opens up the possibility of magnetic control of injected cells into chondral defect area. By applying external magnetic device, previous studies validated the possibility of magnetic manipulation of transplanted cells into target area with IONs labeling.^9,10^ Whether this method can be applied on a much more complex anatomical structure such as knee and hip joints needs further investigation.

In the present study, we aimed to investigate the feasibility of magnetic-targeting intra-articular injection with autologous chondrocytes in rabbit chondral defect model. Amphiphilic polyethylenimine (PEI) prepared by partial alkylation was used for hydrophobic IONs phase transferring.^11^ Rabbit chondrocytes were then labeled with PEI/IONs. The efficiency of PEI/IONs labeling in primary chondrocytes, and its influence on the collagen type-II synthesis were investigated. Later on, PEI/IONs labeled chondrocytes were intra-articular injected, and magnetically targeted to the chondral defect with a magnet implanted into subchondral bone in a rabbit model. Biological behavior of injected chondrocytes and initial repair effect were evaluated by histological analysis.

## Methods

### Primary chondrocytes isolation and characterization

All animal procedures were in accordance with the National Institutes of Health Guide for the Care and Use of Laboratory Animals, and were approved by the Animal Ethics Committee of Third Military Medical University (Army Medical University). 3-month old healthy New Zealand rabbits (1.8–2.0 kg) were purchased from and raised in the experimental animal center of Third Military Medical University. For primary chondrocytes isolation, three mixed gender rabbits were sacrificed by hyperanesthesia with pentobarbital sodium (90 mg kg^−1^). Articular cartilage of knee joint and costochondral junction was excised and disinfected in 75% ethanol for 5 min. Minced cartilage (1 mm^3^ pieces) was then digested at 37 °C in 0.25% of trypsin (Hyclone, USA) for 2 h, and in Dulbecco's modified Eagle's medium (DMEM, Hyclone, USA) containing 0.2% of collagenase type-II for 2 h (Sigma Chemical Co., USA). Undigested tissue fractures were later removed using a 100 μm filter. Primary chondrocytes were isolated using centrifugation at 1000 rpm for 5 min, and re-suspended in DMEM containing 10% fetal bovine serum (Hyclone, USA), 100 U ml^−1^ penicillin (Hyclone, USA), and 100 mg ml^−1^ streptomycin (Hyclone, USA).^12^ Chondrocytes were cultured at 37 °C, 5% CO_2_, and split when reached 90% confluences.

In order to validate the chondrogenic phenotype of isolated chondrocytes, toluidine blue staining^13^ and immunohistochemistry (IHC) staining against collagen type-II were performed on cultured chondrocytes at passage 2. Chondrocytes were washed with ice-cold phosphate buffered saline (PBS), fixed with 4% paraformaldehyde. For toluidine blue staining, cells were incubated in working solution (10% toluidine blue in 1% sodium chloride solution) for 5 min at room temperature (RT), washed with PBS for 3 times, and dehydrated in ethanol. For IHC staining, fixed cells were washed with PBS for 3 times. The activity of endogenous peroxidase was destroyed using 0.3% (w/v) hydrogen peroxide for 30 min at RT. Cells were washed again with PBS for 3 times, and incubated with triton X-100 (Sigma Chemical Co., USA, 0.3% in PBS) for 15 min at RT. Later on, cells were incubated with primary antibody (rabbit anti-collagen II, 1 : 200 dilution, Millipore, Germany) in 1% bovine serum albumin (BSA) in PBS at 4 °C overnight. After being washed with PBS, cells were incubated horseradish peroxidase-conjugated donkey anti-rabbit IgG (1 : 5000 dilution in 1% BSA in PBS) at 37 °C for 20 min. After extensive washing, cells were visualized using a diaminobenzidine (DAB) kit (ZLI-9019, ZSGB-BIO, China) according to the manufacturer's protocols.

### PEI/IONs labeling of chondrocytes and efficiency analysis

PEI/IONs were kindly provided by Professor Hua Ai (National Engineering Research Center for Biomaterials, Sichuan University, Chengdu, China). As described in previous studies,^5,11^ Fe_3_O_4_ nanocrystals with hydrodynamic diameter of 9 ± 2 nm was prepared by mixing Fe(acac)_3_ (1 mmol) with 1,2-hexadecanediol (5 mmol), oleic acid (3 mmol), and oleylamine (3 mmol) in benzyl ether (10 ml) under nitrogen. Followed by heating to reflux (300 °C) for 1 h and cooling to RT. PEI/IONs nanocomposites with concentration of 1.3275 g Fe ml^−1^, positively charged mean zeta potential around 50 mV, and mean hydrodynamic diameter of 79 ± 28 nm, were synthesized by self-assembly of Fe_3_O_4_ nanocrystals with alkylated PEI25k (polymer/IONs mass ratio: 0.2). Chondrocytes (1 × 10^5^ cells) at passage 2 were incubated with DMEM containing different concentrations of PEI/IONs (equivalent of 2, 4, 6, 8, 10, 12 or 14 μg Fe ml^−1^) for different incubation periods (6, 12, 18, 24, and 30 h) at 37 °C, 5% CO_2_. To verify the cell-labeling of PEI/IONs, ferric ion in labeled chondrocytes (6 μg Fe ml^−1^) was stained with Prussian blue at the end of 24 h incubation period. Cells were washed two times with PBS, and one time with PBS containing 10 U ml^−1^ heparin to remove excess positively charged PEI/IONs. Cells were fixed with 4% formaldehyde for 30 min, washed, and incubated for 30 minutes with 2% potassium ferrocyanide in 6% hydrochloric acid. Cell nuclei were counterstained with fast red for cell-counting. Cells were considered positive if intracytoplasmic blue granules were detected. In order to analyze the cell-labeling efficiency of PEI/IONs, 1 × 10^4^ chondrocytes labeled with different concentration of PEI/IONs at different incubation periods were harvested and washed two times with PBS, one time with PBS containing 10 U ml^−1^ heparin, and incubated with cell lysis buffer (Solarbio, Beijing, China); followed by iron content analysis with atomic absorption spectroscopy (BH5100, Bohui, China).

### Transmission electron microscopy study

After 24 h PEI/IONs labeling (6 μg Fe ml^−1^), chondrocytes were harvested, washed three times with PBS and centrifuged at 1000 rpm for 15 min. Cell pellet was fixed in 2.5% buffered glutaraldehyde for 30 min at 4 °C, followed by treatment with 1% osmium tetroxide for 30 min. Samples were dehydrated in a concentration gradient of ethanol, immersed in propylene-oxide, and embedded with Epon 812 (Shell Chemical Co., USA). Samples were then sliced into sections with thickness of 60 μm. Transmission electron microscope pictures were taken with an acceleration voltage of 80 kV (Tecnai-10, Tecnai, USA).

### Influence of PEI/IONs on viability and collagen type-II synthesis of labeled chondrocytes

The effect of PEI/IONs on the viability of labeled chondrocytes was evaluated using WST-1 kit (Sigma Chemical Co., USA).^14^ Chondrocytes seeded in 96-well plate (5000 cells per well) were labeled with PEI/IONs (equivalent of 0, 6, 8 μg Fe ml^−1^, in DMEM containing 10% fetal bovine serum) according to method described above. After 24 h of labeling, cells were washed twice with PBS. Followed by incubation with WST-1 reagent (200 μl per well) at 37 °C for 2 h. The absorbance of supernatant was measured with a plate reader (HTS 7000 Plus Bio assay reader, Perkin Elmer, USA) at 450 nm.

In order to evaluate the influence of PEI/IONs labeling on the collagen type-II synthesis of chondrocytes, expression level of COL2A1 was evaluated by RT-qPCR. Chondrocytes labeled with different concentration of PEI/IONs (0, 6 and 8 μg Fe ml^−1^) for 24 h were collected with 0.25% trypsin (Sigma Chemical Co., USA). The total cell RNA was extracted using RNAiso Plus (Takara Bio, Dalian, Shandong, China) according its manufacturer's instruction. The purity of RNA was measured and quantitated on a Nanodrop-1000 spectrophotometer (Thermo Scientific, USA). 1 μg RNA was used for the first strand cDNA synthesis. GAPDH was used as an internal reference, and target gene primer was designed as follow: COL2A1, forward, 5-ACACTGCCAACGTCCAGATC-3, reverse, 5-GTGATGTTCTGGGAGCCCTC-3. Real-time qPCR was performed in a reaction volume of 25 μl using QuantiTect SYBR Green PCR kit (QIAGEN, USA). Assays were performed in triplicates, and the mRNA levels were normalized to GAPDH using the ΔΔCT method.

### Chondral defect creation and *in vivo* magnetic-targeting animal model

To test the feasibility of magnetic-targeting intra-articular injected autologous chondrocytes in knee joint, eight 3-month old New Zealand rabbits were anesthetized with intravenous pentobarbital sodium (30 mg kg^−1^). The left hind leg was shaved and draped in a sterile fashion. Trochlear groove was exposed by an anteromedial incision. A 5 mm in diameter and 6 mm in depth chondral defect in trochlear groove was created with a self-made annular bone drill. Animals were randomly divided into two groups: magnetic-targeting (*n* = 4) and control (*n* = 4) groups. An NdFeB magnet (0.35 T, 5 mm in diameter, 4 mm in depth, Jinxin, China) was used as the magnet source and implanted into the defect area in magnetic-targeting group; where the defects of subchondral bone were filled with bone cement in control group. In order to distinguish injected chondrocytes from host cells, rabbit chondrocytes at passage 2 were first incubated with BrdU^15^ (10 μg ml^−1^ in DMEM) for 48 h, washed with PBS, and then incubated with PEI/IONs (6 μg Fe ml^−1^) for another 24 h. At 1 week post-surgery, 3 ml of saline containing double-labeled chondrocytes (1 × 10^6^ cells per ml) were transplanted into knee cavity through intra-articular injection for both groups. In order to facilitate cell migration and to increase the intra-articular space, additional 1–2 ml of saline was injected 10 min post first injection. All animals were allowed to move freely post operation.

### Histology

Animals were sacrificed at 1-week post intra-articular injection. Trochlear groove was dissected for macroscopic observation and histological evaluation. For histological evaluations, samples containing the defect area were trimmed, and fixed in 4% formaldehyde solution for 48 h. After the magnet or bone cement was carefully removed from the subchondral bone plate, samples were decalcified in 6% nitric acid for 5–7 days before embedding in paraffin. Serial sections were cut at up to 5 μm thickness and stained with hematoxylin and eosin (H&E) for histological examination.

In order to verify the origin of cells participated the chondral defect repairing, injected chondrocytes were detected with IHC against BrdU, and Prussian blue staining as previously described. To quantitatively analyze the percentage of injected cells in repaired tissue, the percentage of BrdU and Prussian blue positive cells were calculated in randomly chosen 200 μm^2^ areas.

### Statistical analysis

All data were presented as mean ± SD. Statistical analyses were performed with SPSS (version 12.0, IBM, USA). Statistical significance was determined by a one-way ANOVA with Bonferroni's *post hoc* test for iron content, cell viability, and COL2A1 expression level analyses. For percentage of BrdU and Prussian blue positive cells analyses, statistical significance were determined by two-tailed Student *t*-test. A *P*-value of 0.05 indicated statistical significance in all analyses.

## Results

### Cell culture and characterization

**Fig. 1:**
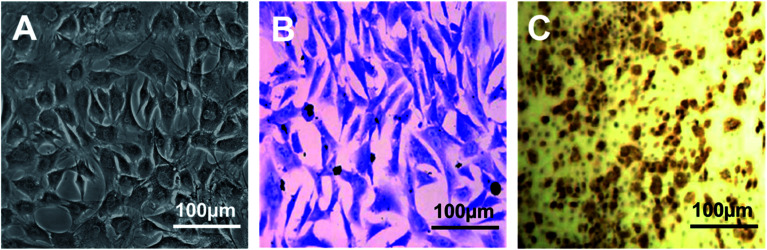
Primary chondrocytes culture and characterization. Cellular morphology of rabbit primary chondrocytes at passage 2 ((A), bright field). Chondrocytes at passage 2 were positively stained by toluidine blue (B) and immunohistochemistry (IHC) against collagen type-II (C).

Cell attachment of isolated rabbit chondrocytes was observed at 2 days culture post tissue digestion. Chondrocytes in primary culture were small and polygonal with high refraction. Cell morphology was remained until passage 2 (Fig. 1A). In later passages such as the third and fourth, chondrocytes appeared larger and more flattened with decreased refraction, indicating dedifferentiation of chondrocytes.^12^ Toluidine blue and IHC staining against collagen type II on chondrocytes showed that cultured chondrocytes at passage 2 were positive for toluidine blue staining and collagen type II staining (Fig. 1B and C).

### PEI/IONs labeling of chondrocytes

**Fig. 2:**
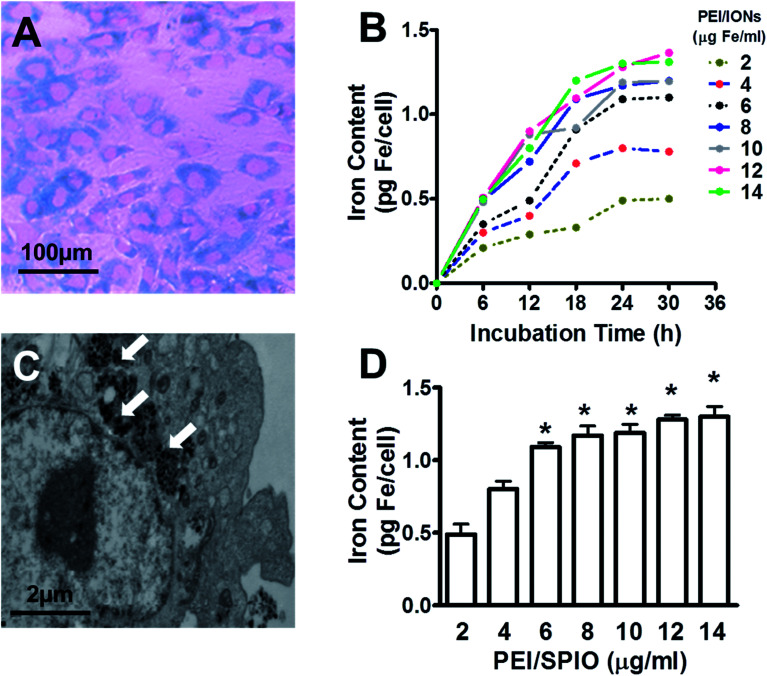
PEI/IONs labeling of rabbit primary chondrocytes. Prussian blue staining (A) of rabbit primary chondrocytes at passage 2 labeled with PEI/IONs (6 μg Fe ml^−1^). Iron content measurement (B) indicated time- and dose-dependent labeling of PEI/IONs in chondrocytes when incubated with different concentrations of PEI/IONs (2, 4, 6, 8, 10, 12 or 14 μg Fe ml^−1^) for different incubation periods (6, 12, 18, 24, and 30 h). Transmission electron microscopy image (C) showed deposits of IONs (white arrows) inside the cytoplasm and vesicles after 24 h incubation with PEI/IONs (6 μg Fe ml^−1^). (D) Compared with 2 μg ml^−1^ group, iron content at 24 h showed significantly higher intracellular iron content in 6, 8, 10, 12, and 14 μg ml^−1^ groups (*P < 0.05, *n* = 6).

Chondrocytes were able to be directly labeled with PEI/IONs by adding probes to the culture medium after 24 h incubation with a final iron concentration of 6 μg ml^−1^. Results showed that most chondrocytes were positive for Prussian blue staining (Fig. 2A). Iron-containing sites presented as blue vesicles or particles in the cytoplasm. Transmission electron microscopy results showed deposits of nanoparticles inside the cytoplasm and vesicles (Fig. 2C). As shown in Fig. 2B, labeling efficiency analysis by measuring iron content suggested time- and dose-dependent labeling manner of PEI/IONs in chondrocytes. PEI/IONs uptake process in chondrocytes occurred most within 24 h in all concentrations. Longer incubation time did not significantly increase intracellular iron content. Compared with 2 μg ml^−1^ group, iron content assay at 24 h showed significantly higher intracellular iron content in 6, 8, 10, 12, and 14 μg ml^−1^ groups (*P* < 0.05). No significant difference in iron content was found among the rest groups.

### Influence of PEI/IONs on viability and collagen type-II synthesis of labeled chondrocytes

**Fig. 3:**
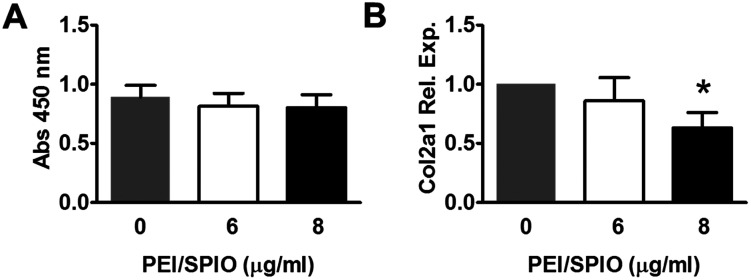
Influences of PEI/IONs on cell viability and collagen type-II synthesis of labeled chondrocytes. WST-1 assay (A) at 24 h showed no significant decreases in viability of PEI/IONs-labeled chondrocytes in all concentration groups compared with that of unlabeled (0 μg ml^−1^) cells (*P > 0.05). RT-qPCR assay of COL2A1 expression level (B) at 24 h showed no significant difference among unlabeled and 6 μg ml^−1^ groups. However, significant decrease of COL2A1 expression was found in 8 μg ml^−1^ group when compared with unlabeled group (*P < 0.05, *n* = 5–6).

As shown in Fig. 3A, WST-1 assay at 24 h showed no significant decreases in viability of PEI/IONs -labeled chondrocytes in 6 or 8 μg ml^−1^ groups when compared with that of unlabeled cells over 24 h (*P* > 0.05). RT-qPCR assay of COL2A1 expression level at 24 h showed no significant difference among unlabeled and 6 μg ml^−1^ labeled groups. However, significant decrease of COL2A1 expression was found in 8 μg ml^−1^ labeled group when compared with unlabeled group (Fig. 3B, *P* < 0.05).

### *In vivo* magnetic-targeting of PEI/IONs labeled chondrocytes

**Fig. 4:**
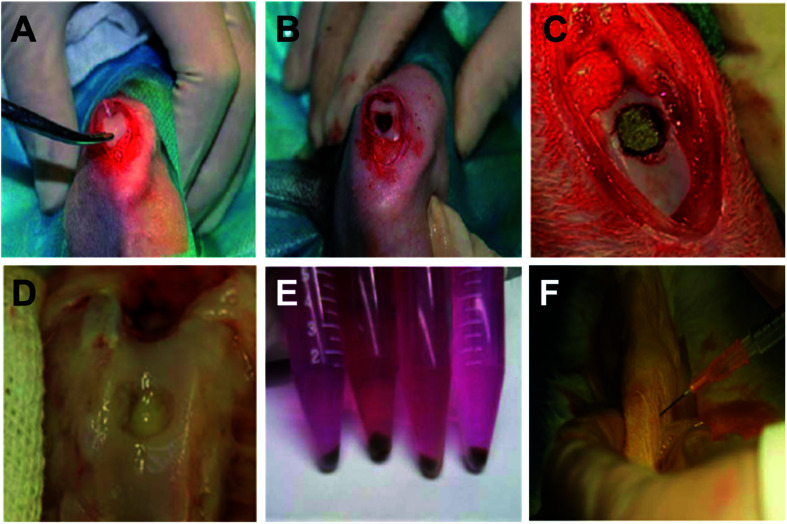
*In vivo* magnetic-targeting of PEI/IONs labeled chondrocytes in experimental rabbit model. The creation process of chondral defect in trochlear groove (A and B) and surgical implantation of magnet (C) or bone cement (D) for magnetic-targeting and control groups, respectively. PEI/IONs and BrdU double labeled chondrocytes (E) were intra-articular injected in both groups (F).

Fig. 4A–D showed the creation of chondral defect in trochlear groove and surgical implantation of magnet or bone cement for magnetic-targeting and control groups, respectively. Animals were allowed to move freely post operation. As shown in Fig. 4E and F, PEI/IONs and BrdU double-labeled chondrocytes were transplanted into knee cavity through intra-articular injection in both groups. Animals were healthy throughout the whole post-implantation period except one animal in control group dropped out the study due to unknown infection.

**Fig. 5:**
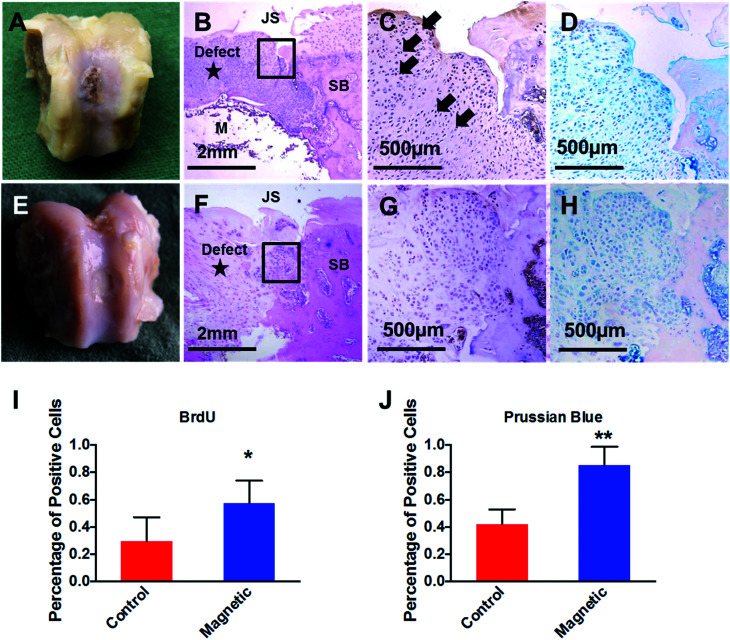
Macrographic observation and histological evaluation of magnetic-targeting outcome in the knee joint of experimental rabbit model at 1 week post intra-articular injection. Macrographic observation (A and E) showed that chondral defect was filled by IONs-labled chongdrocytes in magnetic-targeting group (A). Hematoxylin and eosin (H&E) staining (B) indicated chondrocytes aggregation above implanted magnet when compared with same region in control group (F). JS, joint surface; SB, subchondral bone; M, implanted magnet. IHC staining against BrdU (C and G, higher-power magnification of the boxed area in B and F) and respective quantitative results (I) demonstrated higher density of injected BrdU positive chondrocytes (black arrows) in magnetic-targeting group. In addition, compared with control group (H), Prussian blue staining (D and H) and quantitative results (J) at the same regions of magnetic-targeting group (D) suggested higher percentage of IONs labeled chondrocytes (*P < 0.05, **P < 0.01, *n* = 6).

By the time of necropsy at 1 week post intra-articular injection, no significant inflammation was observed in the knee joint cavity. Synovial fluid remained transparent. Macroscopic observation showed that chondral defect remained unrepaired at 2 week post operation in both groups (Fig. 5A and E). However, as shown in Fig. 5B and F, histology examination with H&E suggested higher density of chondrocytes aggregated at the defect region when compared with adjacent native articular cartilage tissue. Compared with control group, higher density of BrdU positive chondrocytes were observed in magnetic-targeting group (Fig. 5C and G). In addition, Prussian blue staining (D&H) suggested higher percentage of PEI/IONs positive chondrocytes at the same regions in magnetic-targeting group.

## Discussion

Cell-based therapy is promising in OA clinical intervention. Compared with traditional methods such as bone marrow stimulation or autologous cartilage implantation, cell-based therapy through intra-articular injection has been shown to be safe and efficient in reducing OA symptoms.^2,3^ However, due to the complex anatomical structure and physiological environment of joint cavities, none-targeting cell delivery during the injection process, and cells adhesion to peripheral tissues could cause adverse effects such as synovial hyperplasia and formation of scare tissue.^16,17^ Magnetic-labeling and targeting could play an important role in reducing these side effects. Our previous study using large animal model has shown the feasibility of IONs in cell tracking under MRI scanning.^18^ In addition, the potential of magnetic-targeting of IONs labeled chondrocytes has been tested in our preliminary research utilizing *ex vivo* chondral defect in human total knee arthroplasty samples.^19^ In the present study, we showed convenient magnetic-labeling of PEI/IONs when co-cultured with primary chondrocytes, while the collagen type-II synthesis of labeled chondrocytes was unaffected at lower concentration. Furthermore, our preliminary animal study using rabbit chondral defect model showed superior cell delivery of PEI/IONs labeled chondrocytes when magnetic-targeting was utilized.

The clinically approved IONs were traditionally used as contrast agents that enable MRI with shortened T1 and T2 relaxation times.^20^ Due to the negative or non-charge surface, traditional IONs were suffered from weak cytoplasm internalization in nonphagocytic cells without the presence of cationic liposomes.^21,22^ In the present study, the amphiphilic PEI with partial alkylation modification was used for phase transferring of hydrophobic IONs, and to avoid nanocrystal aggregation and preserve similar magnetic property of IONs in organic solvents at the same time.^11^ With co-culture method, our *in vitro* data here showed time- and dose-dependent labeling of PEI/IONs in primary chondrocytes. Convenient labeling of PEI/IONs was able to achieve at concentration of 6 μg Fe ml^−1^. Furthermore, similar to traditional IONs,^22^ our results revealed no effect of PEI/IONs labeling on the viability of chondrocytes within 24 h. However, suppressed collagen type-II synthesis was observed in 8 μg ml^−1^ group, indicating carefully considered dosage of PEI/IONs for chondrocyte in further application. In addition, the long-term cytotoxicity and chondrogenic suppression of PEI/IONs on chondrocytes still remains unclear. Cytotoxicity in high-molecular weight PEI has been suggested due to higher number of amine groups on the polymer.^23^ Further modification such as lactosylation on PEI might help with the cytotoxicity reduction and remain the magnetic property of IONs.^24^

Recently, the potential of magnetic nanoparticles in tissue regeneration has been studied in many tissue types.^25–28^ Using magnetic nanoparticles coated scaffolds, Panseri *et al.* showed early restoration of complex osseous structure in rabbit bone defect model.^29^ In addition, the feasibility of magnetically guided endothelial cells and MSCs delivery post stent implantation,^30–32^ and its in-stent stenosis prevention effect have been also recently demonstrated.^33^ Herein, to validate the *in vivo* potential in magnetic-targeting of PEI/IONs in knee joint, PEI/IONs and BrdU double labeled chondrocytes were injected into chondral defect knee joint. Histology data at the defect site demonstrated that the injected chondrocytes aggregated at the defect area in the presence of magnetic force. In contrast, lower density of BrdU and Prussian blue positive chondrocytes in the same region of control group suggesting none-targeting distribution of injected chondrocytes.^16^ In addition, our data suggested that adhesion of magnetic targeted chondrocytes was secure, and was not influenced by the daily movement of knee joint. Although the long term effect of magnetic force on chondrocyte phenotype needs further study, magnetic-targeting of PEI/IONs cells could reduce the drawbacks like surgical trauma, risk of periosteum detachment and heterotopic ossification in previous methods for cell adhesion.^34–37^

There are several limitations of this study that necessitate discussion. First, the present study is a preliminary *in vivo* study in order to test the feasibility of magnetic-targeting of PEI/IONs labeled cells in complex anatomical knee joint. Parameters such as field density, traction force and targeting time of implanted magnetic device were not included. Second, implanted magnet was used as a compromised source of magnetic force in our study. Surgical trauma and inflammatory reaction could to some extent influence the histologic evaluation and long-term repair outcome. Despite the immune reaction caused by implanted magnet, ferric ion release from magnet degradation could be absorbed by chondrocytes and thus cause a higher percentage of Prussian blue positive cells when compared with the percentage of BrdU positive cells during image quantification. A well-designed external magnetic device seems necessary for further investigation. Third, the *in vivo* observation period was relatively short. Although our data from mammalian toxicology examination at 3 month of rabbit subcutaneously injected PEI/IONs suggested negative impact on the animal's internal organs (heart, kidney, and liver; Fig. 1). The cytotoxicity of alkylation modified PEI post PEI/IONs labeling in prolonged *in vitro* culture, and the long-term *in vivo* repair outcome of magnetic-targeting of PEI/IONs labeled cells for chondral defect should be addressed in our future study.

## Conclusion

Despite these limitations, the present study showed proof of concept experiments in magnetic-targeted autologous chondrocytes intra-articular injection for chondral defect repair. Our method provided new opportunity to improve the targeting of implanted cells during current cartilage repair strategies such as autologous chondrocyte implantation (ACI), autologous bone marrow derived mesenchymal stem cells (BMSCs)^38^ and autologous adipose derived mesenchymal stem cells (ADSCs)^39^ injection. In these clinical treatments, magnetic labeling could be conveniently applied on isolated cells during operation, and postoperatively injected under the magnetic guidance from well-designed magnetic source, which might ultimately lead to an improved repair outcome.

## Conflicts of interest

There are no conflicts to declare.

## Supplementary Material

RA-008-C7RA12039G-s001
