# Supplementary material for: Magnetic-targeting of polyethylenimine-wrapped iron oxide nanoparticle labeled chondrocytes in a rabbit articular cartilage defect model
Source: RSC Adv. 2018 Feb 16;8(14):7633–40. doi: 10.1039/c7ra12039g (PMC9078383; doi:10.1039/c7ra12039g)
Supplement: RA-008-C7RA12039G-s001 [file RA-008-C7RA12039G-s001.pdf]

Electronic Supplementary Material (ESI) for RSC Advances.
This journal is © The Royal Society of Chemistry 2018

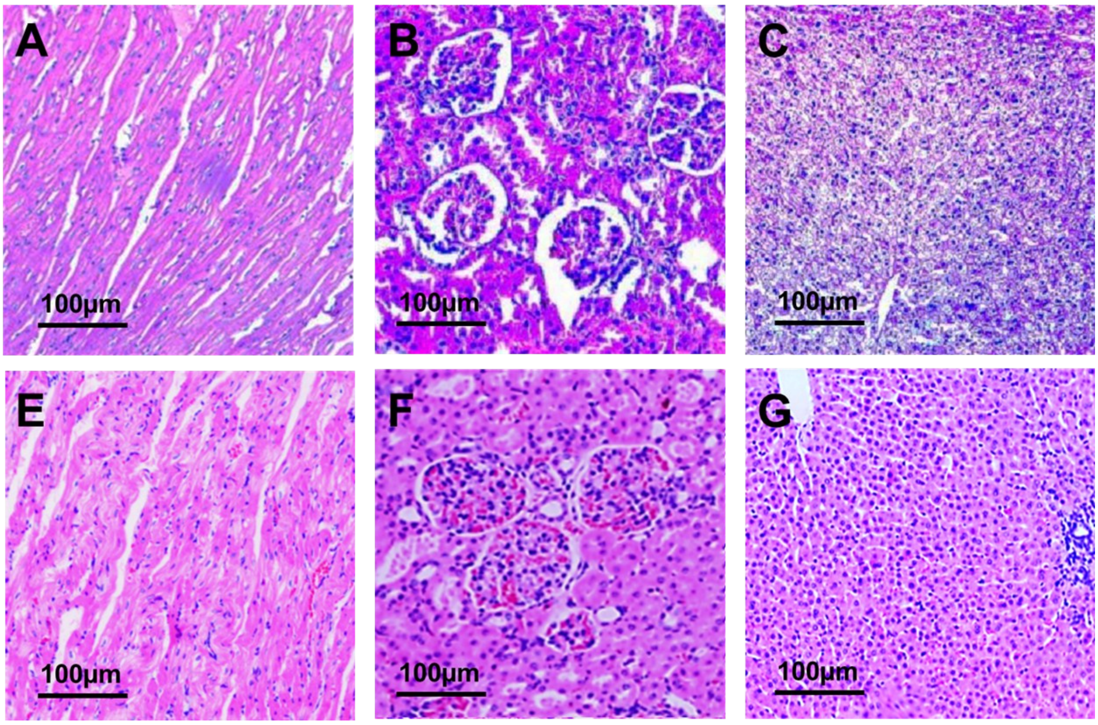

Supplementary Fig. 1 Toxicity examination results on the animal's internal organ from magnetic-targeting and control groups. Representative images of H&E staining for heart (A&E), kidney (B&F), and liver (C&G) of animal in magnetic-targeting group (upper panel) and control group (lower panel).
